# Reactive oxygen species (ROS)-responsive biomaterials for treating myocardial ischemia-reperfusion injury

**DOI:** 10.3389/fbioe.2024.1469393

**Published:** 2024-09-02

**Authors:** Ying Zhang, Mantang Jiang, Tao Wang

**Affiliations:** ^1^ Natural and Biomimetic Medicine Research Center, Tissue-Orientated Property of Chinese Medicine Key Laboratory of Sichuan Province, West China Hospital, Sichuan University, Chengdu, China; ^2^ Institute of Integrated Traditional Chinese and Western Medicine, West China Hospital, Sichuan University, Chengdu, China

**Keywords:** myocardial ischemia-reperfusion injury, ROS-responsive biomaterials, nanoparticals, hydrogels, biomimetic biomaterials

## Abstract

Myocardial ischemia-reperfusion injury (MIRI) is a critical issue that arises when restoring blood flow after an ischemic event in the heart. Excessive reactive oxygen species (ROS) production during this process exacerbates cellular damage and impairs cardiac function. Recent therapeutic strategies have focused on leveraging the ROS microenvironment to design targeted drug delivery systems. ROS-responsive biomaterials have emerged as promising candidates, offering enhanced therapeutic efficacy with reduced systemic adverse effects. This review examines the mechanisms of ROS overproduction during myocardial ischemia-reperfusion and summarizes significant advancements in ROS-responsive biomaterials for MIRI treatment. We discuss various chemical strategies to impart ROS sensitivity to these materials, emphasizing ROS-induced solubility switches and degradation mechanisms. Additionally, we highlight various ROS-responsive therapeutic platforms, such as nanoparticles and hydrogels, and their unique advantages in drug delivery for MIRI. Preclinical studies demonstrating the efficacy of these materials in mitigating MIRI in animal models are reviewed, alongside their mechanisms of action and potential clinical implications. We also address the challenges and future prospects of translating these state of the art biomaterial-based therapeutics into clinical practice to improve MIRI management and cardiac outcomes. This review will provide valuable insights for researchers and clinicians working on novel therapeutic strategies for MIRI intervention.

## 1 Introduction

Myocardial infarction (MI) remains the leading cause of morbidity and mortality worldwide, underscoring the urgent need for innovative therapeutic strategies (Reed et al., 2017; Laforgia et al., 2022). MI is characterized by the occlusion of coronary arteries, resulting in a deprivation of oxygen and nutrients to the damaged myocardium. Re-establishing blood flow through reperfusion, typically via percutaneous coronary intervention or thrombolysis, is critical for saving the ischemic myocardium (Thygesen et al., 2007; Saito et al., 2023). However, this process induces a series of harmful effects, including oxidative stress caused by a surge in reactive oxygen species (ROS), calcium overload, and a strong inflammatory response, leading to further myocardial damage, known as myocardial ischemia-reperfusion injury (MIRI) (Yellon and Hausenloy, 2007; Algoet et al., 2023; Liu Y. et al., 2023).

ROS, including superoxide anion (O_2_^•−^), hydroxyl radical (^•^OH), and hydrogen peroxide (H_2_O_2_), are natural byproducts of cellular metabolism, primarily generated in mitochondria during oxidative phosphorylation (Sies et al., 2022). Under normal physiological conditions, ROS are tightly regulated by endogenous antioxidant systems, maintaining redox homeostasis. However, during myocardial I/R, the abrupt reintroduction of oxygen during reperfusion disrupt the balance between ROS production and scavenging, resulting in an uncontrolled burst of ROS from various sources, including the mitochondria, xanthine oxidase, NADPH oxidases, and uncoupled nitric oxide synthase (NOS). This excessive ROS generation triggers a cascade of deleterious events, including lipid peroxidation, protein oxidation, DNA damage, inflammation, and various forms of programmed cell death (e.g., apoptosis, pyroptosis, ferroptosis), ultimately exacerbating myocardial injury and dysfunction (Cadenas, 2018; Dubois-Deruy et al., 2020).

Over the past few decades, significant progress has been made in developing anti-MIRI drugs (Ibanez et al., 2015; Zhou et al., 2021), however, systemic drug administration faces challenges such as poor targeting, limited efficacy, and potential toxic side effects. Recent advancements in nanotechnology and biomaterials have paved the way for creating targeted therapeutic strategies that deliver drugs specifically to ischemia-reperfusion injured myocardium (Luo et al., 2023). The unique ischemia-reperfusion microenvironment (IME), characterized by acidosis, elevated ROS levels, and massive inflammatory cell infiltration, has become a critical target for developing controlled-release strategies for anti-MIRI drugs (Hausenloy and Yellon, 2013; Li and Gao, 2023).

Among the various strategies targeting the IME, ROS-responsive nanomaterials are currently the most advanced and promising. These materials are engineered to specifically detect abnormal ROS levels at the site of injury and to trigger the controlled release of therapeutic agents or modulation of cellular signaling pathways, thereby improving tissue inflammation (Chung et al., 2015; Lee et al., 2018; Xia et al., 2023), impaired angiogenesis (Chen et al., 2021; Zheng et al., 2022a), or fibrosis (Surendran et al., 2020; Gaytan et al., 2023; Zhang J. et al., 2024). Strategies for fabricating ROS-responsive biomaterials encompass a diverse array of approaches, primarily based on ROS-induced solubility switches and degradation mechanisms (Lee S. H. et al., 2013; Xu et al., 2016b). Solubility switching strategies exploit the reversible transformation of the amphiphilic nature of materials towards ROS, thereby achieving controlled release of the encapsulated therapeutic agent. In contrast, the ROS-induced degradation strategy involves incorporating ROS-labile linkages within the polymer backbone, designed to cleave upon exposure to ROS, leading to material degradation and subsequent release of the therapeutic agent. Biomaterials that utilize ROS-induced solubility switching or degradation elements, including polymeric nanoparticles, injectable hydrogels, and biomimetic nanoparticles, loaded with anti-MIRI drugs possessing antioxidant, anti-inflammatory, pro-survival, or pro-angiogenic properties, have become promising targeted therapeutic strategies for MIRI in the past decade (Bae et al., 2016; Ziegler et al., 2019; Li et al., 2020; Li et al., 2021; Hao T. et al., 2022; Hou et al., 2022; Huang et al., 2022; Lan et al., 2022; Li et al., 2022; Weng et al., 2022; Zhang X. et al., 2022). These ROS-responsive nanomedicines have demonstrated their precise targeting and controlled drug release capabilities and achieved enhanced therapeutic effects in MIRI animal models, providing strong support for their future clinical applications.

**FIGURE 1:**
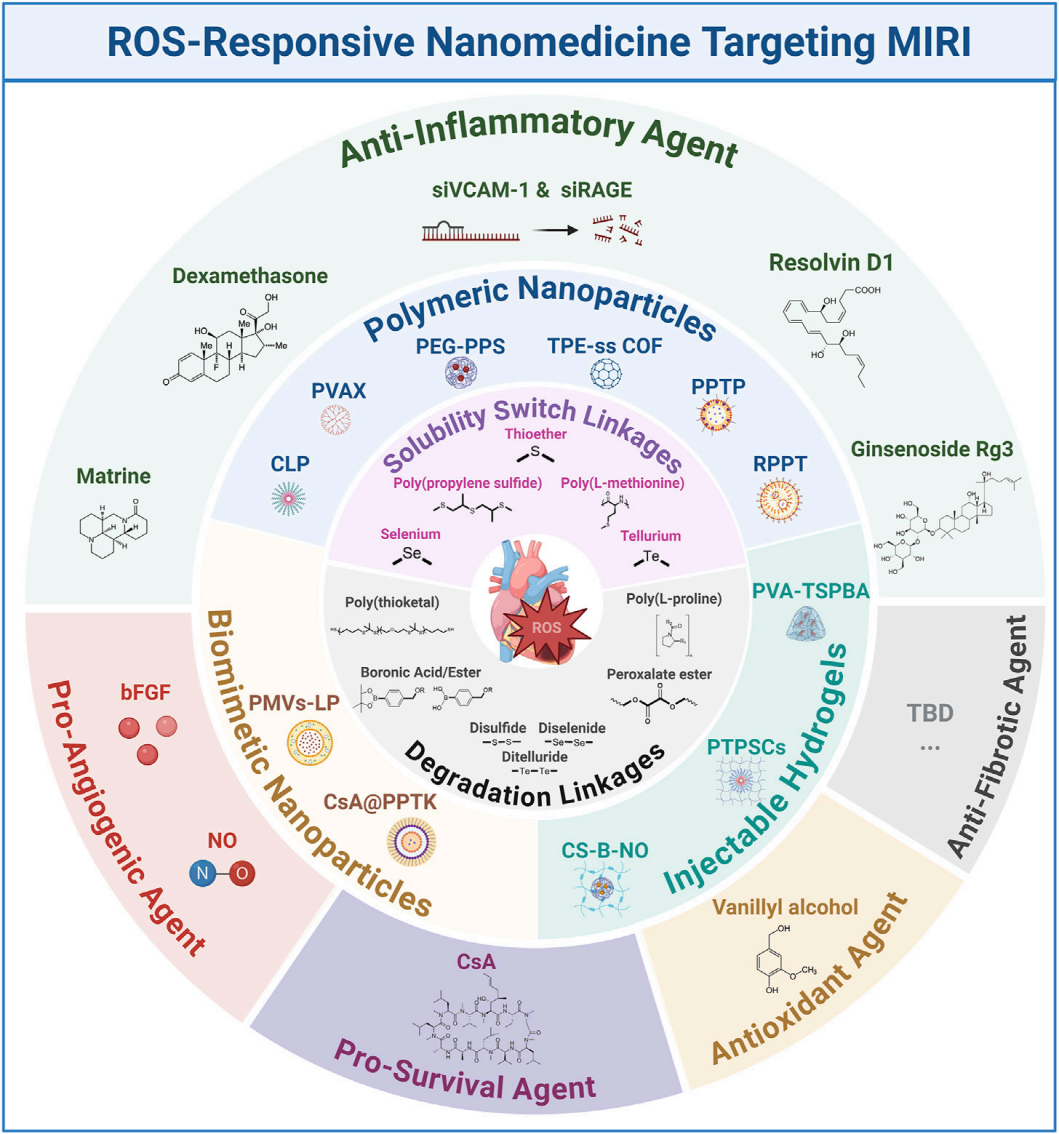
Schematic representation of ROS-responsive nanomedicines targeting MIRI. bFGF: basic fibroblast growth factor; COF: covalent organic framework; CLP: an amphiphilic copolymer was designed and synthesized by sequentially conjugating luminol and PEG with Ce6; CS-B-NO: chitosan modified by boronate-protected diazeniumdiolate; LP: liposome; NO: nitric oxide; PPTP: PGE_2_-PEG modified tellurium-crosslinked polyethyleneimine; PEG: poly (ethylene glycol); PPS: poly (propylene sulfide); PMVs: platelet mem brane vesicles; PVA: poly (vinyl alcohol); PTPSCs: PLGA-TK-PEG-SS31; TBD: to be determined; TPE: Tetraphenylethene; TSPBA: N1-(4-boronobenzyl)-N3-(4-boronophenyl)-N1,N1,N3,N3-tetramethylpropane-1,3-diaminium.

In this review, we aim to provide a comprehensive overview of the progress in ROS-responsive biomaterials for treating MIRI over the past decade (Figure 1). We summarize the mechanisms underlying ROS overproduction during myocardial I/R, strategies for fabricating ROS-responsive biomaterials, and the current ROS-responsive therapeutic platforms for MIRI intervention. We also discuss the challenges and future prospects of translating these technologies into clinical practice, aiming to improve cardiovascular health and patient outcomes.

## 2 ROS overproduction during myocardial I/R

**FIGURE 2:**
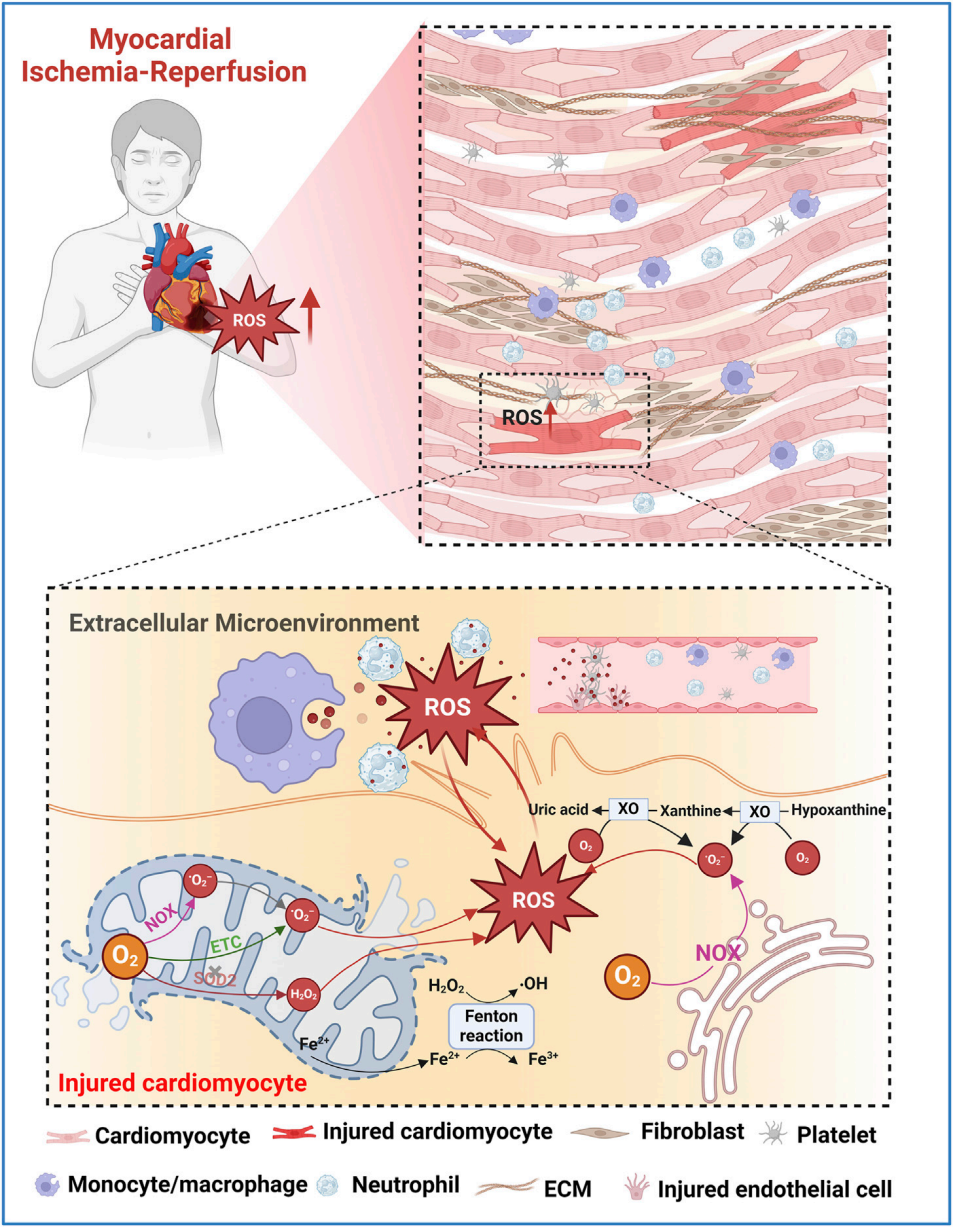
Schematic illustration of potential ROS sources in injured myocardium during MIRI. O_2_^•−^ are primarily produced in the mitochondrial ETC. Fe^2+^ released by the ruptured mitochondria generates •OH via Fenton reaction. Mitochondrial NOX also generates O_2_^•−^. Damaged antioxidant systems, such as SOD2, fail to adequately remove O_2_^•−^ or H_2_O_2_ leading to the accumulation of ROS. In the extramitochondrial space, NOX and XO play major roles in ROS production during I/R. Excessive ROS causes oxidative damage to lipids, proteins, and nucleic acids, leading to cell death and exacerbating I/R injury. The increased oxidative stress in the extracellular microenvironment of the injured myocardium is mainly attributed to the contents released after the rupture of the plasma membrane of damaged cardiomyocytes, as well as the ROS released by neutrophils, macrophages, endothelial cells, and activated platelets.

The excessive generation of ROS is a pivotal factor in MIRI. This ROS storm triggers a cascade of deleterious events, including mitochondrial dysfunction and lipid peroxidation, leading to cardiomyocyte necrosis and various forms of programmed cell death such as apoptosis, pyroptosis, and ferroptosis (Cadenas, 2018; Gong et al., 2024). Several recent reviews have well elucidated the mechanisms of ROS generation following myocardial I/R and its complex role in MIRI (Cadenas, 2018; Bugger and Pfeil, 2020). Here, we focus on providing a summary of current knowledge about the source and mechanism of ROS overproduction after myocardial I/R from the perspective of different cell types in damaged myocardium, including cardiomyocytes and non-cardiomyocytes (e.g., inflammatory cells, endothelial cells, and platelets). (Figure 2).

### 2.1 ROS generation in cardiomyocytes

#### 2.1.1 Mitochondrial electron transport chain (ETC)

Mitochondria are the primary sources of ROS production in damaged cardiomyocytes during myocardial I/R (Bugger and Pfeil, 2020). The ETC complexes, particularly complexes I and III, are key sites where electron leakage occurs, leading to the formation of superoxide anion (O_2_^•−^). During ischemia, the activity of complex I is inhibited due to oxygen depletion, causing electron accumulation. Upon reperfusion, these electrons react with the reintroduced oxygen, generating a significant amount of O_2_^•−^ through reverse electron transport (RET) (Paradies et al., 2004; Chen et al., 2008; Vinogradov and Grivennikova, 2016). This process is exacerbated by the accumulation of succinate during ischemia, which is rapidly oxidized upon reperfusion, driving RET and enhancing ROS production (Chouchani et al., 2014). Complex II also contributes to ROS generation, though to a lesser extent than complexes I and III (Chouchani et al., 2014; Chouchani et al., 2016). Additionally, Fe^2+^ released by the ruptured mitochondria reacts with intracellular H_2_O_2_, triggering the Fenton reaction and generating the highly strong oxidant •OH (Gordan et al., 2018).

#### 2.1.2 NADPH oxidase (NOX)

NADPH oxidases are another critical source of ROS in both cardiomyocytes and non-cardiomyocytes (Kahles and Brandes, 2013). NOX enzymes, particularly NOX2 and NOX4, are upregulated in response to I/R injury (Braunersreuther et al., 2013). These enzymes transfer electrons from NADPH to oxygen, producing O_2_^•−^. NOX-derived ROS are implicated in various pathophysiological processes, including endothelial dysfunction and inflammation (Paravicini and Touyz, 2008). Inhibition of NOX activity has been shown to reduce myocardial infarct size and ROS levels (Thirunavukkarasu et al., 2012; Braunersreuther et al., 2013), underscoring its significance in I/R injury.

#### 2.1.3 Xanthine oxidase (XO)

Xanthine oxidase is a key enzyme in purine metabolism that generates ROS as a byproduct in cardiomyocytes (Boueiz et al., 2008). During ischemia, xanthine dehydrogenase is converted to XO, which then produces O_2_^•−^ and H_2_O_2_ during reperfusion. XO-derived ROS contribute significantly to oxidative stress and tissue damage in MIRI (Madesh and Hajnoczky, 2001; Hool, 2009). The use of XO inhibitors, such as allopurinol, has demonstrated cardioprotective effects by reducing ROS production and subsequent cellular injury (Grimaldi-Bensouda et al., 2015).

### 2.2 ROS generation in endothelial cells (ECs)

ECs are pivotal in the pathophysiology of MIRI and other cardiovascular diseases, largely due to their role as major sources of ROS (Yang et al., 2016; Chen et al., 2019; Haybar et al., 2019). Among the key mechanisms regulated by ECs is the synthesis of nitric oxide (NO), a vasodilator with protective cardiovascular effects, mediated by endothelial nitric oxide synthase (eNOS) (Mount et al., 2007). Under physiological conditions, eNOS efficiently produces NO, which not only promotes vascular relaxation but also exerts antioxidant effects. However, during oxidative stress, the cofactor tetrahydrobiopterin (BH4), essential for eNOS function, undergoes oxidation, leading to the uncoupling of eNOS. This uncoupling diverts eNOS activity from NO production to the generation of O_2_^•−^, thereby increasing ROS levels and exacerbating endothelial dysfunction (Dumitrescu et al., 2007; Alkaitis and Crabtree, 2012). Strategies to recouple eNOS, such as supplementation with BH4 or its precursors, have shown promise in reducing ROS production and ameliorating cardiac damage (Antoniades et al., 2006). Despite the antioxidant role of NO under normal conditions, its interaction with O_2_^•−^ can result in the formation of peroxynitrite (ONOO^−^), a harmful oxidant (Radi, 2013). The presence of ONOO^−^ further intensifies oxidative stress, contributing to greater myocardial damage during IR injury (Liu et al., 2005; Yu et al., 2018).

### 2.3 ROS generation in inflammatory cells

Inflammatory cells, such as neutrophils and macrophages, are recruited to the site of injury during reperfusion. These cells produce large amounts of ROS via their NADPH oxidase systems in response to inflammatory stimuli (Chakraborti et al., 2000). Neutrophil-derived ROS contribute to tissue damage by exacerbating oxidative stress and promoting further recruitment of inflammatory cells (Jordan et al., 1999). The interaction between inflammatory cells and damaged endothelium also creates a vicious cycle of ROS production and cellular injury (Knock, 2019).

### 2.4 ROS generation in activated platelets

Platelets also contribute to ROS production after myocardial I/R. During I/R, platelets become activated and release ROS through their mitochondrial pathways and NADPH oxidase systems (Begonja et al., 2005). Platelet-derived ROS also plays a significant role in amplifying the inflammatory response and promoting thrombus formation, which can further obstruct blood flow and exacerbate myocardial injury (Takaya et al., 2005; Xu et al., 2006). Targeting platelet activation has shown potential in reducing the extent of myocardial damage and improving reperfusion outcomes (Schanze et al., 2023).

Collectively, the overproduction of ROS during myocardial I/R injury arises from various cellular sources, including cardiomyocytes, endothelial cells, inflammatory cells, and platelets. Functional impairment or activity changes of mitochondrial ETC, NADPH oxidase, XO and uncoupled eNOS during I/R are the core regulatory mechanisms leading to excessive ROS generation in different cell types in the injured myocardium. Understanding these sources and their mechanisms provides critical insights into the pathophysiology of MIRI. Future efforts to design ROS-responsive biomaterials should focus on targeting the specific features of excessive ROS production in the myocardial IME, with an emphasis on developing innovative biomaterials with precise cell type-specific and subcellular organelle-targeting (e.g, mitochondria) properties.

## 3 Strategies for ROS-responsive biomaterials fabrication

The emergence and widespread application of ROS-responsive biomaterials have greatly changed the limitations of current anti-MIRI drug interventions. These advanced materials are designed to engage proactively with the pathophysiological oxidative milieu, offering a targeted and controlled release of therapeutic agents. This section delves into the mainstream strategies for the fabrication of ROS-responsive biomaterials, focusing on ROS-induced solubility switching and degradation mechanisms (Table 1).

**TABLE 1:** ROS-responsive elements.

ROS-responsive mechanism	ROS-responsive elements		Chemical structure and oxidation	Sensitivity	References
Solubility Switch	Sulfur-Based linkages	Thioether	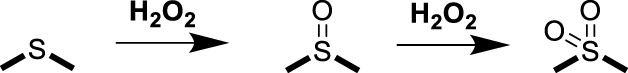 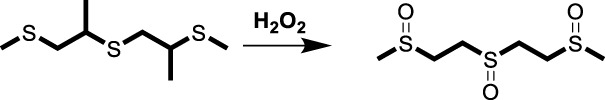 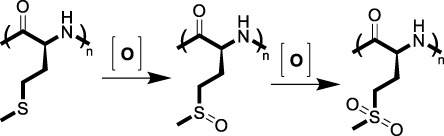	H_2_O_2_: 3.3 vol%	Gruhlke and Slusarenko (2012), Xu et al. (2016a), Wang et al. (2022), Yue et al. (2023), Yang et al. (2024)
		Poly (propylene sulfide)		H_2_O_2_ : 100 μM	
		Poly (L-methionine)		H_2_O_2_ : 1 mM	
	Selenium-linkages		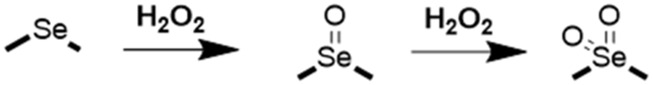	H_2_O_2_ : 0.1% v/v	Ma et al. (2010), Tan et al. (2012), Xu et al. (2013)
	Tellurium- linkages		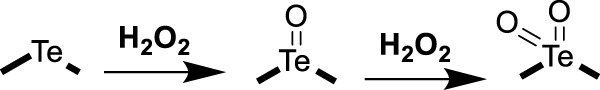	H_2_O_2_ : 100 μM	Cao et al. (2015), Wang et al. (2015)
Degradation	Phenylboronic Acid and Ester		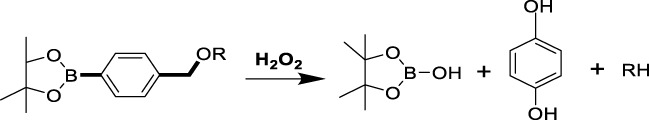 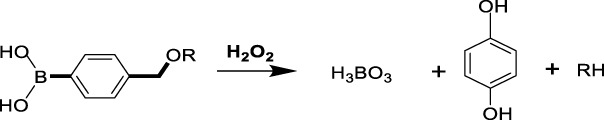	H_2_O_2_ : 50 μM	Sun et al. (2013), Stubelius et al. (2019), Han and Domaille (2022)
	Poly (thioketal)		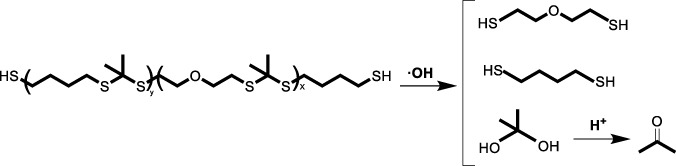	H_2_O_2_ : 0.2% v/v	Liu and Thayumanavan (2020), Wang et al. (2020), Xie et al. (2022), Yao et al. (2022b), Shen et al. (2024)
	Peroxalate ester		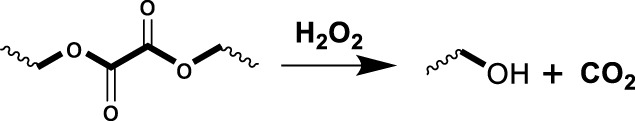	H_2_O_2_: >50 nM	Romanyuk et al. (2017)
	Poly (L-proline)		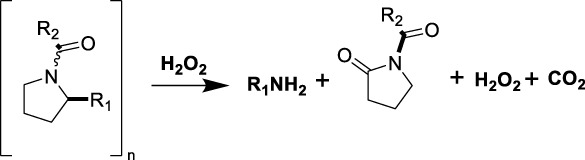	H_2_O_2_ : 5 mM	Yu et al. (2011), Lee et al. (2014), Gupta et al. (2015)
	Disulfide, diselenide, and ditelluride bond		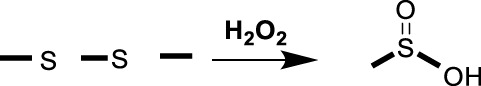 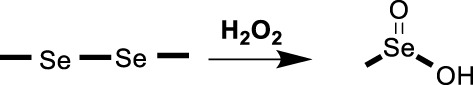 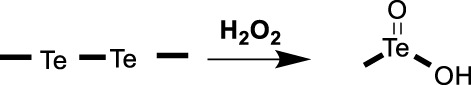	H_2_O_2_: <20 μM	Hou et al. (2022), Lan et al. (2022), Cao et al. (2024), Kang et al. (2024)

### 3.1 ROS-induced solubility switch

The principal strategy for fabricating ROS-responsive biomaterials involves the incorporation of functional groups that undergo significant solubility changes in response to ROS. Under oxidative conditions, these groups typically transform from hydrophobic to hydrophilic states, enhancing water solubility and facilitating the release of encapsulated drugs.

#### 3.1.1 Sulfur-containing polymers

Sulfur-containing polymers are among the earliest developed ROS-responsive systems (Yue et al., 2023). Due to sulfur’s diverse oxidation states, ranging from −2 to +6, these materials can undergo significant changes upon exposure to ROS. When oxidized, sulfide-containing polymers form sulfoxides or sulfones, increasing their hydrophilicity. This change leads to polymer swelling, disassembly, and eventual drug release (Gruhlke and Slusarenko, 2012). Poly (propylene sulfide) (PPS) is the most fundamental used sulfur-containing ROS-responsive functional group. PPS-based nanoparticles, stable in water, degrade rapidly upon exposure to H_2_O_2_ but not to superoxide, releasing encapsulated drugs effectively (Shofolawe-Bakare et al., 2022; Bezold et al., 2023). Thioether is another well-studied ROS-responsive linker that undergoes a hydrophobic to hydrophilic transition upon oxidation, which makes thioether-containing polymers widely used in the precise delivery and release of drugs in response to oxidative microenvironment-related diseases (Wang et al., 2022). Additionally, poly (L-methionine), an amino acid-based polymer, exhibits ROS-responsive behavior through the oxidation of its sulfur-containing methionine residues to sulfoxides and sulfones. This oxidation alters the polymer’s hydrophobicity, leading to structural changes and the release of therapeutic agents (Xu et al., 2016a; Yang et al., 2024). Given the inherent advantages of this amino acid-based polymer, such as biocompatibility and biodegradability, it may become a key material in the design of next-generation therapeutics for the treatment of ROS-related diseases, such as cancer and chronic liver disease (Yoo et al., 2017; Hao Y. M. et al., 2022).

#### 3.1.2 Selenium-containing polymers

Selenium-based materials exhibit a more pronounced response to ROS compared to sulfur-based ones due to selenium’s lower electronegativity and larger atomic radius (Xu et al., 2013). These materials can undergo a solubility switch due to the oxidation of selenium moieties to selenoxides or selenones upon exposure to low concentrations of H_2_O_2_ (0.01% v/v) (Ma et al., 2010), leading to the release of encapsulated therapeutic agents. The development of selenium-containing block copolymers, such as PEG-PUSe-PEG, has shown promise in drug delivery applications where the hydrophobic polyurethane block containing selenides can self-assemble into micelles that disassemble upon ROS exposure (Tan et al., 2012). In addition, the biological significance of selenium, particularly its role in enhancing the catalytic activity of glutathione peroxidase (GPx), makes it an ideal candidate for the design of ROS-responsive materials (Kieliszek and Blazejak, 2013).

#### 3.1.3 Tellurium-containing polymers

Tellurium, positioned below selenium in the periodic table, offers even higher sensitivity to ROS due to its chemical properties, making tellurium-containing polymers ultra-responsive and suitable for applications in pathological microenvironment with relative low levels of ROS (Cao et al., 2015; Wang et al., 2015). Similar to selenium-based delivery systems, tellurium-containing biomaterials exhibit pronounced solubility changes under oxidative conditions (Wang et al., 2015), ensuring timely and controlled drug release. Their potential in therapeutic applications, particularly in inflammation and tumor progression sites (Lu et al., 2017; Dominguez-Alvarez et al., 2022), is being explored, although research in this area remains limited.

### 3.2 ROS-induced degradation

Another critical strategy involves the design of biomaterials that degrade upon exposure to ROS through the cleavage of specific chemical bonds within the polymer backbone, thereby releasing the therapeutic agents in a controlled manner. This degradation mechanism is pivotal for the functionality of ROS-responsive biomaterials in targeted drug delivery and tissue engineering applications.

#### 3.2.1 Phenylboronic acid and ester-containing polymers

Phenylboronic acid and its ester derivatives are exceptionally sensitive to ROS, undergoing rapid oxidative degradation due to the cleavage of the boron-carbon bond (Han and Domaille, 2022). Under oxidizing conditions, the linkage between boronic acids/esters and the material or drug molecules of interest becomes oxidized with the insertion of oxygen (Stubelius et al., 2019). Notably, the reaction kinetics are significantly influenced by the nucleophilicity of the boron center, with nucleophilic boronic esters reacting more quickly than their corresponding acids (Sun et al., 2013). Moreover, recent studies have shown that boronic esters with ether bonds exhibit excellent degradation kinetics at biologically relevant concentrations of H_2_O_2_, around 50 μM (Jourden et al., 2011). This sensitivity makes boronic ester-containing polymers some of the most ROS-responsive materials available, particularly suitable for applications requiring high sensitivity to ROS and precise control over the release of therapeutic agents.

Interestingly, most of currently reported anti-MIRI hydrogel drug delivery systems that employ ROS-responsive degradation mechanisms incorporate boronic acid or boronic ester as their core ROS-responsive functional groups (detailed in Section 4) (Li et al., 2021; Hao T. et al., 2022; Zhang X. et al., 2022). This preference is likely due to their proven effectiveness in maintaining hydrogel stability while allowing for precisely controlled degradation and drug release in response to oxidative stress.

#### 3.2.2 Poly (thioketal) (TK) polymers

Similar to boronic esters, thioketal linkages are destabilized in the presence of O_2_^•−^and H_2_O_2_, leading to the oxidation into ketones and organic thiols or disulfides (Liu and Thayumanavan, 2020). This degradation mechanism has been widely used to develop ROS-responsive materials containing thioketals for targeted drug release therapy in diseases characterized by high ROS microenvironments, such as enteritis (Shen et al., 2024), wound repair (Martin et al., 2014), and ischemic heart disease (Xie et al., 2022; Yao Y. et al., 2022).

#### 3.2.3 Peroxalate ester-containing polymers

Peroxalate esters are another class of ROS-responsive polymers that degrade in the presence of relative low H_2_O_2_ (>50 nM), producing carbon dioxide and other byproducts, with the potential to generate chemiluminescence (Romanyuk et al., 2017; Hao Y. M. et al., 2022). This characteristic makes them particularly valuable in applications that benefit from real-time monitoring of ROS levels, as the chemiluminescence can serve as an optical signal for the presence of oxidative stress.

In the context of MIRI, peroxalate ester-containing polymers have shown potential in both diagnostic and therapeutic roles. The generated luminescent signals can help in the detection of ROS bursts during reperfusion, while the degradation of the polymer allows for the controlled release of therapeutic agents precisely when ROS levels are elevated (Lee D. et al., 2013). Recent advancements in the design of peroxalate ester-based hydrogels have also demonstrated their capability to act as both ROS scavengers and drug delivery platforms, thereby providing a dual function in mitigating oxidative damage and delivering cardioprotective drugs (Bae et al., 2016).

#### 3.2.4 Poly (L-proline)-containing polymers

Poly (L-proline) is another naturally occurring amino acid-based polymer that has shown particularly sensitive to ROS due to the presence of pyrrolidine rings (Tian et al., 2021). Under oxidative stress, the proline residue undergoes oxidation, leading to the cleavage of the polymer backbone (Amici et al., 1989). This degradation results in the release of entrapped drugs or therapeutic agents, making poly (L-proline) a useful material in drug delivery systems, particularly for applications where controlled degradation is essential (Dai et al., 2023). Poly (L-proline) has been widely used in the fabrication of scaffolds for tissue engineering applications where the degradation rate can be matched to the tissue regeneration process (Yu et al., 2011; Lee et al., 2014). The ROS-responsiveness of these scaffolds can be further enhanced by incorporating additional ROS-sensitive elements such as boronic esters or thioketal groups.

#### 3.2.5 Disulfide, diselenide, and ditelluride bond-bontaining polymers

Compared with the integration of individual chalcogen elements (sulfur, selenium, tellurium) to impart solubility transition properties to nanomaterials, recent studies have increasingly favored the use of disulfide, diselenide, and ditelluride bonds as ROS-responsive degradation elements (Yuan et al., 2021; Hou et al., 2022; Lan et al., 2022; Weng et al., 2022; Qiu et al., 2024). These bonds spontaneously cleave under oxidative conditions, leading to the degradation of nanomaterials and enabling controlled drug release.

Disulfide bond (-S-S-) is widely used due to their stability under normal conditions and their ability to break in the presence of ROS, such as H_2_O_2_ (as low as 20 μM), making them ideal for delivery systems for tumors and diseases related to ischemia-reperfusion injury (Kang et al., 2024; Qiu et al., 2024). Diselenide bond (-Se-Se-), with lower bond dissociation energy than disulfides, responds more quickly to ROS and have been explored in neurodegenerative diseases and MIRI (Yuan et al., 2021; Weng et al., 2022). Although less studied, the ditellurium bond (-Te-Te-) is the most sensitive to ROS and can respond rapidly, and has recently been increasingly studied for its application in nanomaterial design to alleviate diseases such as MIRI where excessive ROS production is present (Hou et al., 2022; Lan et al., 2022).

## 4 ROS-responsive therapeutic platforms for MIRI intervention

In recent decades, ROS-responsive functional groups have been extensively integrated into various biomaterials, such as polymer nanoparticles, hydrogels, patches, and biomimetic materials (Zhang et al., 2015; Xu et al., 2016a; Chakraborty et al., 2023). These advancements enable targeted and controlled drug release at sites with elevated ROS levels. Given the excessive ROS production in damaged myocardium following ischemia-reperfusion injury (Cadenas, 2018), recent developments in nanomedicine have increasingly utilized these ROS-responsive elements to achieve precise, targeted delivery of cardioprotective drugs with anti-inflammatory (Li et al., 2020; Hou et al., 2022; Huang et al., 2022; Lan et al., 2022; Weng et al., 2022), antioxidant (Bae et al., 2016), pro-survival (Li et al., 2022; Zhang X. et al., 2022), or pro-angiogenic properties (Li et al., 2021; Hao T. et al., 2022). By responding to ROS stimuli, these drugs can be released specifically at the site of myocardial injury, enhancing therapeutic efficacy while minimizing side effects.

Although several recent reviews have comprehensively explored the landscape of ROS-related nanoplatforms for alleviating MIRI, their main focus is on ROS-scavenging nanozymes and nanomaterials loaded with antioxidant drugs (Zhang Z. et al., 2022; Li et al., 2023). In contrast, here we focus on the application of various ROS-responsive biomaterials in alleviating MIRI in the past decade. Additionally, we systematically summarize the preferred ROS-responsive functional groups used in these biomaterials and highlight the cardioprotective properties of the encapsulated drugs.

### 4.1 Polymeric nanoparticles

Biocompatible and versatile polymers such as poly (lactide-co-glycolide) (PLGA), poly (ethylene glycol) (PEG), and polyethylenimine (PEI) are commonly used to create multifunctional drug-loaded nanoparticles (Sah et al., 2013). To achieve ROS-responsive drug release, these nanoparticles often incorporate ROS-sensitive functional groups as linkers either between polymeric backbone chains or between the polymer and the drug molecule. Upon exposure to elevated ROS levels, these linkers undergo degradation or solubility changes, triggering the controlled release of the encapsulated drugs (Xu et al., 2016b). Currently, sulfide and tellurium moieties are particularly prevalent as ROS-responsive components in polymeric nanoparticles designed to selectively respond to the oxidative stress in the injured myocardium.

#### 4.1.1 Sulfide-based linkages

**FIGURE 3:**
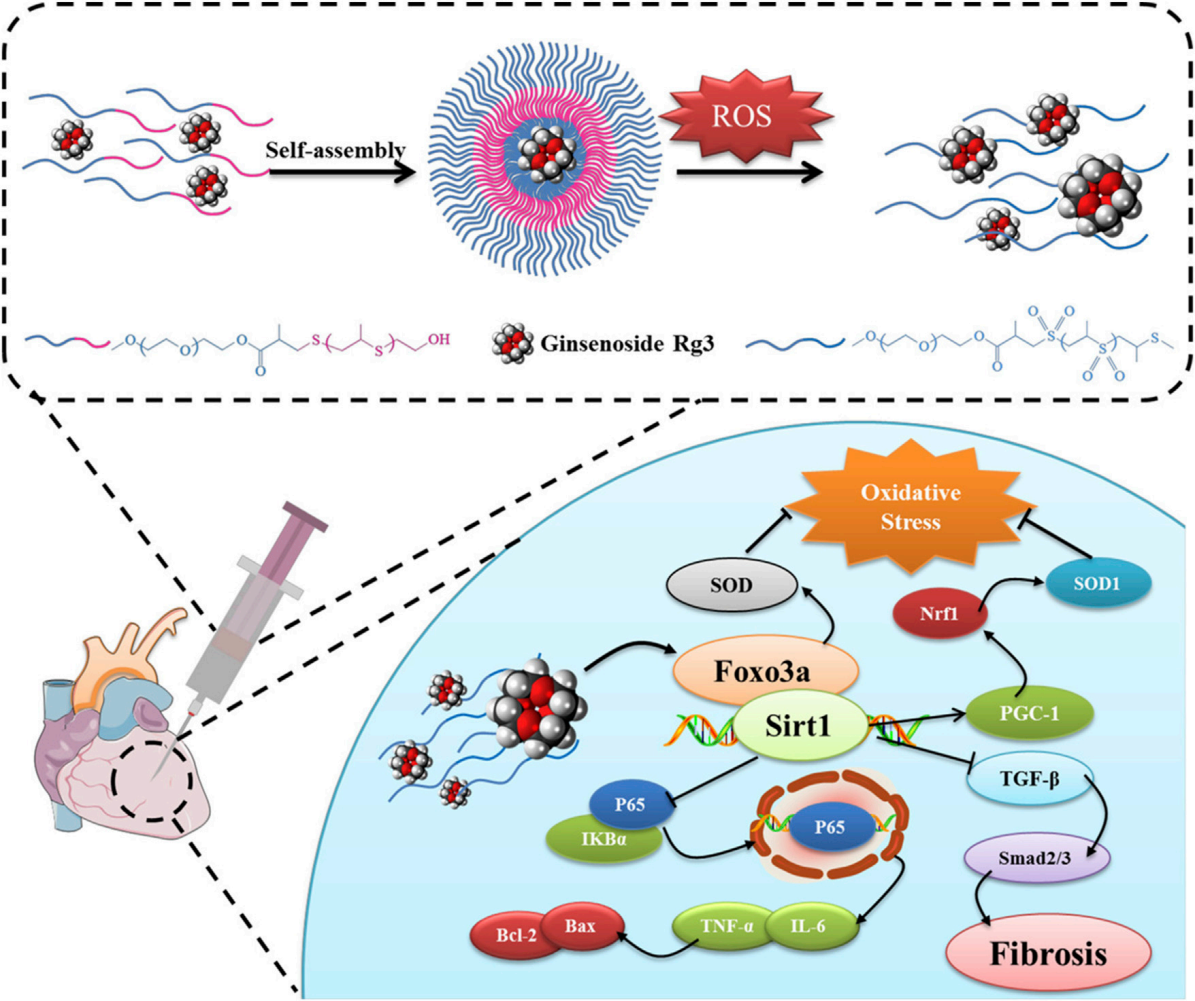
ROS-responsive polymeric nanoparticles were created through the self-assembly of diblock copolymers of poly (ethylene glycol) (PEG) and poly (propylene sulfide) (PPS) and used to encapsulate and deliver Rg3 to sites of MIRI. Upon intramyocardial injection, the Rg3-loaded PEG-b-PPS nanoparticles responded to ROS, releasing Rg3, which then mitigated MIRI by interacting with FoxO3a, exerting anti-oxidative, anti-inflammatory, and anti-fibrotic effects. Reproduced with permission (Li et al., 2020). Copyright 2019, Elsevier.

Li and colleagues have developed a ROS-responsive polymeric nanoparticle synthesized from diblock copolymers of PEG and PPS for the delivery of ginsenoside Rg3, a natural product with potent antioxidant properties, to mitigate MIRI (Li et al., 2020). Upon encountering ROS, the sulfide linkages within the PPS undergoes oxidative conversion from a hydrophobe to a hydrophile, releasing Rg3 specifically at the site of injury. In a rat model of MIRI, the intramyocardial injection of these nanoparticles demonstrated improved cardiac function and reduced infarct size (Figure 3). The therapeutic action of Rg3 was found to be mediated through activating FoxO3a, a protein involved in oxidative stress regulation (Link, 2019).

Distinct from sulfide-based ROS-responsive solubility transitions, Huang et al. developed a biodegradable, redox-responsive covalent organic framework (COF) nanocarrier integrating tetraphenylethene (TPE) and disulfide moieties (ss) for prolonged MIRI therapy (Huang et al., 2022). The TPE-ss COF system demonstrated exceptional redox-responsiveness, degrading efficiently in the presence of H_2_O_2_ and facilitating the controlled release of therapeutic agent matrine, a natural quinolizidine alkaloid that protects cells from ischemia-reperfusion injury by attenuating c-Jun N-terminal kinase (JNK) signaling. In rat models, intravenous administration of TPE-ss COF@Matrine markedly reduced myocardial infarction area, enhanced cardiac function, and alleviated myocardial fibrosis and cardiomyocyte apoptosis. Additionally, the nanocarrier exhibited prolonged retention in cardiac tissue, enabling sustained drug delivery.

#### 4.1.2 Ditellurium linkages

**FIGURE 4:**
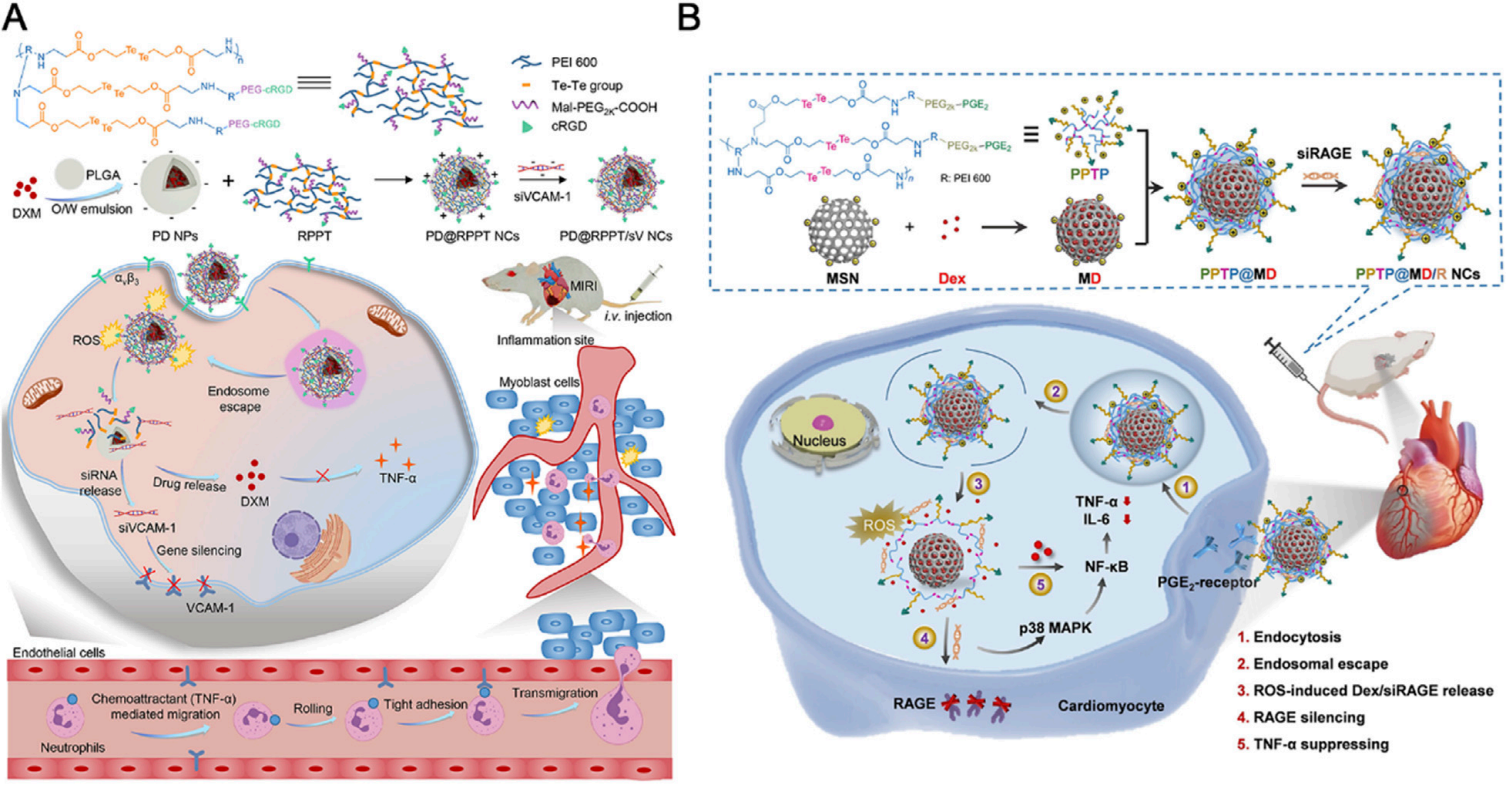
**(A)** The endothelial cell-targeting, ROS-ultrasensitive NCs for co-delivery of siVCAM-1 and DXM aim to treat MIRI inflammation. The ROS-cleavable RPPT, containing ditellurium, was designed to enclose DXM-loaded PLGA NPs and siVCAM-1. After i. v. injection into rats with MIRI, RPPT@siVCAM-1 NCs efficiently targeted inflamed endothelial cells by binding to over-expressed αvβ3 receptors. In the ROS-rich cytoplasm, RPPT degraded, releasing siVCAM-1, enhancing VCAM-1 silencing. This reduced neutrophil recruitment to the injured myocardium, significantly improving anti-inflammatory efficacy and myocardial function recovery. Reproduced with permission (Hou et al., 2022). Copyright 2022, Elsevier. **(B)** Cardiomyocyte-targeted nanotherapeutics for ROS-sensitive co-delivery of siRAGE and Dex were developed. Dex-loaded MSNs, coated with PPTP (a ditellurium-containing polycation), complexed with siRAGE and gated the MSNs to prevent Dex pre-leakage. After systemic injection into myocardial IR-injured rats, the nanotherapeutics entered inflamed cardiomyocytes via PGE2 recognition of over-expressed EP receptors. Intracellular ROS degraded PPTP, releasing siRAGE and Dex to silence RAGE and manage myocardial inflammation. Reproduced with permission (Lan et al., 2022). Copyright 2022, Tsinghua University Press.

Tellurium’s superior responsiveness to ROS makes it a valuable component in polymeric nanoparticle design, facilitating the controlled delivery of encapsulated drugs by regulating material’s solubility changes under oxidative conditions (Cao et al., 2015). For instance, Hou and colleagues have engineered an endothelial cell-targeting and ROS-ultrasensitive nanocomplex system for the co-delivery of dexamethasone (DXM) and VCAM-1 siRNA (siVCAM-1) to treat MIRI (Hou et al., 2022). The nanocomplexes, termed RPPT, were synthesized by crosslinking PEI with ditellurium and subsequently modified with PEG and the endothelial cell-targeting peptide cRGD (Figure 4A). Upon systemic administration in a rat model of MIRI, the cRGD-modified nanocomplexes efficiently targeted and entered the inflamed endothelial cells located in the injured myocardium. There, RPPT was sensitively degraded by overproduced ROS, triggering the release of intracellular siVCAM-1 and DXM, thereby effectively abolishing the expression of the neutrophil recruiter VCAM-1 and inhibiting the production of proinflammatory factors such as TNF-α. The combined action of DXM and siVCAM-1 cooperatively inhibited both migration and adhesion of neutrophils, effectively mitigating the inflammatory response and reducing MIRI.

Interestingly, the same research team used a similar design strategy to create an anti-inflammatory nanocomplex that specifically targets inflamed cardiomyocytes to combat MIRI (Lan et al., 2022). This ROS-responsive nanocomplex was synthesized by conjugating PEGylated prostaglandin E2 (PGE_2_-PEG) with ditellurium-crosslinked polyethyleneimine (PEI), which was then coated with DXM and loaded with receptor for advanced glycation end-products (RAGE) siRNA (siRAGE) onto mesoporous silica nanoparticles (MSNs) (Figure 4B). The resulting nanocomplex exhibited high stability in serum, preventing premature degradation of siRNA and enabling efficient ROS-responsive release of siRAGE, achieving a 72% RAGE silencing efficiency, along with the delivery of DXM within inflamed cardiomyocytes. When administered intravenously to MIRI rats, this nanocomplex significantly reduced myocardial inflammation, leading to substantial improvements in myocardial function and reduced fibrosis.

#### 4.1.3 Others

In addition to the commonly used chalcogen compounds mentioned above, peroxalate ester linkages with H₂O₂ sensitivity and scavenging capabilities have been utilized to develop ROS-responsive nanoparticles for treating MIRI (Bae et al., 2016). Specifically, Bae and colleagues designed H₂O₂-responsive antioxidant polymer nanoparticles, known as PVAX, by incorporating peroxalate ester linkages with the naturally occurring antioxidant compound vanillyl alcohol (VA) in their backbone. These nanoparticles were synthesized using an emulsion/solvent evaporation method. The PVAX polymer rapidly degraded at sites of ROS overproduction, demonstrating superior therapeutic effects by reducing myocardial infarction size and apoptosis through its potent antioxidant properties.

Moreover, with the breakthrough development of fluorescent nanoprobes in the field of precise disease diagnosis, there have been continuous studies in recent years to design environmentally responsive fluorescent nanoprobes to respond to specific stimuli (such as ROS) to achieve simultaneous high-contrast imaging and targeted treatment of lesion areas (Shen et al., 2021; Xu et al., 2022; Liu J. et al., 2023; Sun et al., 2023). For example, in the study of Ziegler et al., a self-assembled fluorescent nanoprobe was developed for imaging and therapy of MIRI. This nanoprobe is composed of an amphiphilic copolymer that incorporates a hydrophilic chain of PEG and hydrophobic components of luminol-conjugated chlorin e6 (Ce6) (Ziegler et al., 2019). The unique design allows the nanoprobe to self-assemble into nanoparticles that can specifically target areas of injured myocardium due to the local increase in ROS. The nanoprobe demonstrated high specificity for the ischemic/reperfused myocardium with fluorescence signals up to 24 h post reperfusion in a mouse model of myocardial I/R. Moreover, this study further discusses the broader implications of using ROS-responsive nanoprobe for targeted drug delivery in other ROS-associated conditions such as stroke, renal infarction, and inflammatory diseases. This dual functionality-imaging and therapy-makes fluorescent nanoprobes an attractive platform for the development of theranostics, which combine diagnostics with therapy.

### 4.2 Injectable hydrogels

Hydrogels are high molecular weight polymer materials with a three-dimensional cross-linked network structure, known for their exceptional water absorption and swelling properties, making them excellent drug delivery carriers (Hamidi et al., 2008). Injectable hydrogels, in particular, have emerged as a versatile platform in nanomedicine, offering several advantages in drug delivery and tissue engineering (Bae et al., 2013). They can facilitate local treatment through minimally invasive administration, reducing systemic side effects and improving patient compliance. These hydrogels can be engineered to respond to specific microenvironmental triggers, such as pH, temperature, and oxidative stress, allowing for precise control of drug release and enhancing therapeutic efficacy (Abdollahiyan et al., 2020).

By taking advantage of the high concentration of ROS in the damaged myocardium after I/R, in recent years, there have been continuous studies on the development of ROS-responsive hydrogels to achieve precise targeting and sustained release of drugs by intramyocardial or intrapericardial injection in the damaged area (Li et al., 2021; Hao T. et al., 2022; Zhang X. et al., 2022). These hydrogel designs all use similar ROS-responsive degradation elements, namely boronic acid/ester functional groups. The preference for boronic acid/ester as ROS-responsive elements in hydrogel design may stem from their stability under normal physiological conditions but superior reactivity to elevated ROS levels and ability to undergo rapid degradation to facilitate the precise release of encapsulated therapeutic agents in the injured myocardium.

**FIGURE 5:**
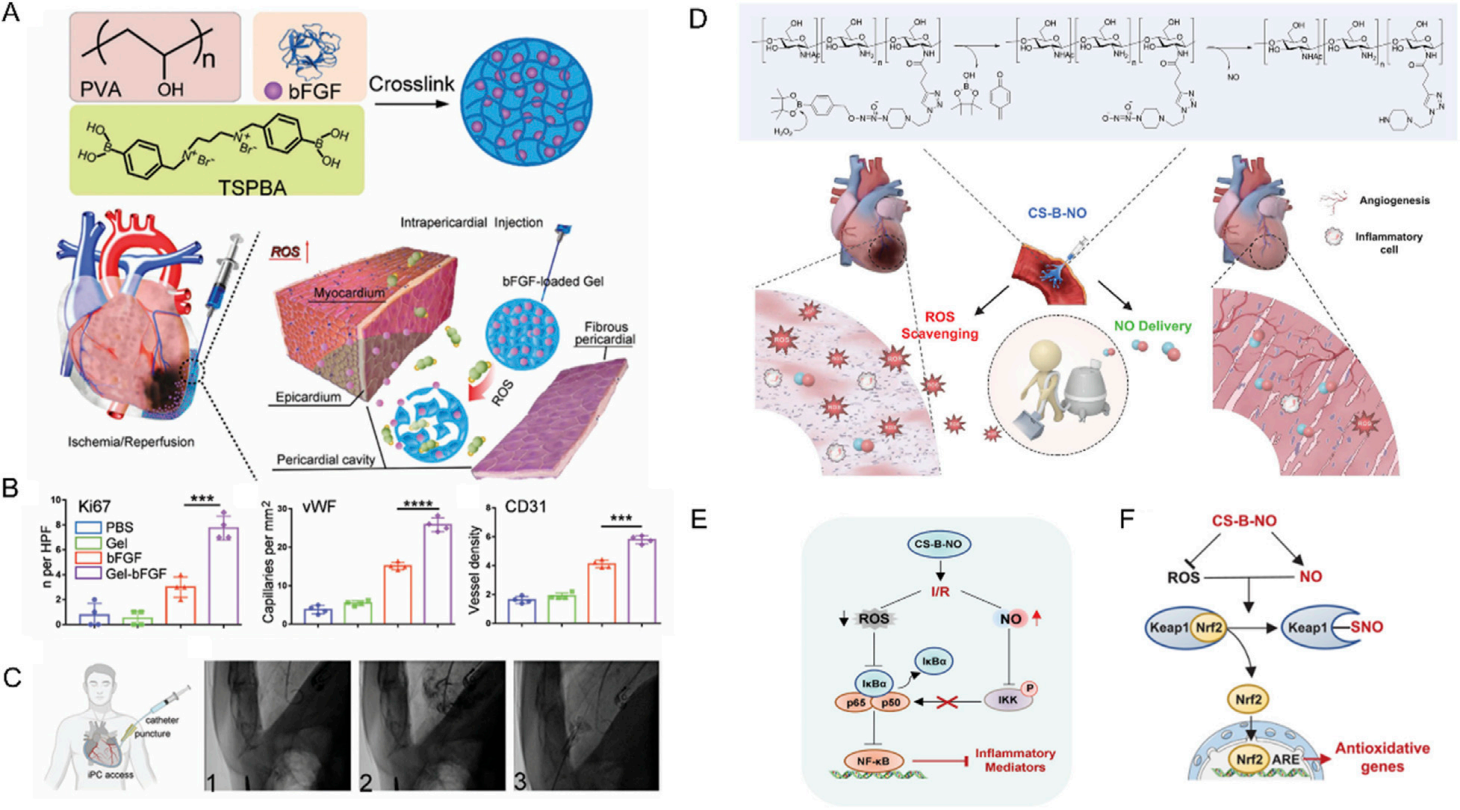
**(A)** Schematic illustration of Gel-bFGF fabrication and overall strategy. **(B)** Quantitative data corresponding to ki67, vWF CD 31 staining showed that Gel-bFGF injection promoted angiomyogenesis. **(C)** A schematic illustrating minimally invasive iPC access in human patients alongside fluoroscopy images from a patient who underwent a LARIAT procedure. Reproduced with permission (Li et al., 2021). Copyright 2021, Wiley. **(D)** Schematic illustration of the treatment of I/R heart injury by CS-B-NO. **(E)** Schematic illustration summarizing the mechanism of CS-B-NO hydrogel on inhibiting the NF-𝜅B signaling pathway after I/R injury. **(F)** Schematic illustration summarizing the mechanism of CS-B-NO hydrogel on activation of the Nrf2 pathway agains oxidative stress via enhancing Keap1 S-nitrosylation. Reproduced with permission (Hao T. et al., 2022). Copyright 2022, Wiley.

For example, Li and colleagues have engineered a ROS-responsive hydrogel loaded with basic fibroblast growth factor (bFGF) for myocardial repair following I/R injury (Li et al., 2021). This innovative hydrogel was synthesized from poly (vinyl alcohol) (PVA) cross-linked with a ROS-sensitive benzoboric acid derivative, enabling the delivery of bFGF directly to the heart’s surface upon injection into the pericardial cavity (iPC) (Figure 5A). This design takes advantage of the elevated ROS levels in the damaged myocardium to trigger “on-demand” release of bFGF, thereby facilitating angiogenesis and enhancing cardiac function in a rat model of I/R injury (Figure 5B). Most importantly, these researchers also proved the feasibility of minimally invasive iPC access in a human patient during a standard LARIAT procedure, highlighting the potential for clinical translation (Figure 5C).

Similarly, Hao and colleagues utilized boronic ester as ROS-responsive element to fabricate an innovative, injectable dual-function hydrogel, CS-B-NO, designed to counteract MIRI by addressing the ROS/NO disequilibrium (Hao T. et al., 2022). This hydrogel, synthesized from chitosan modified with boronate-protected diazeniumdiolate, stands out for its ability to release NO in response to ROS stimulation, thereby modulating the ROS/NO imbalance post-I/R injury (Figure 5D). The CS-B-NO hydrogel exhibited significant therapeutic effects in attenuating cardiac injury and adverse cardiac remodeling in a mouse model of MIRI. The underlying mechanism involved the activation of the antioxidant defense system and protection against oxidative stress induced by I/R injury through the adaptive regulation of the Nrf2-Keap1 pathway, leading to a reduction in inflammation by inhibiting the activation of NF-κB signaling (Bellezza et al., 2018).

**FIGURE 6:**
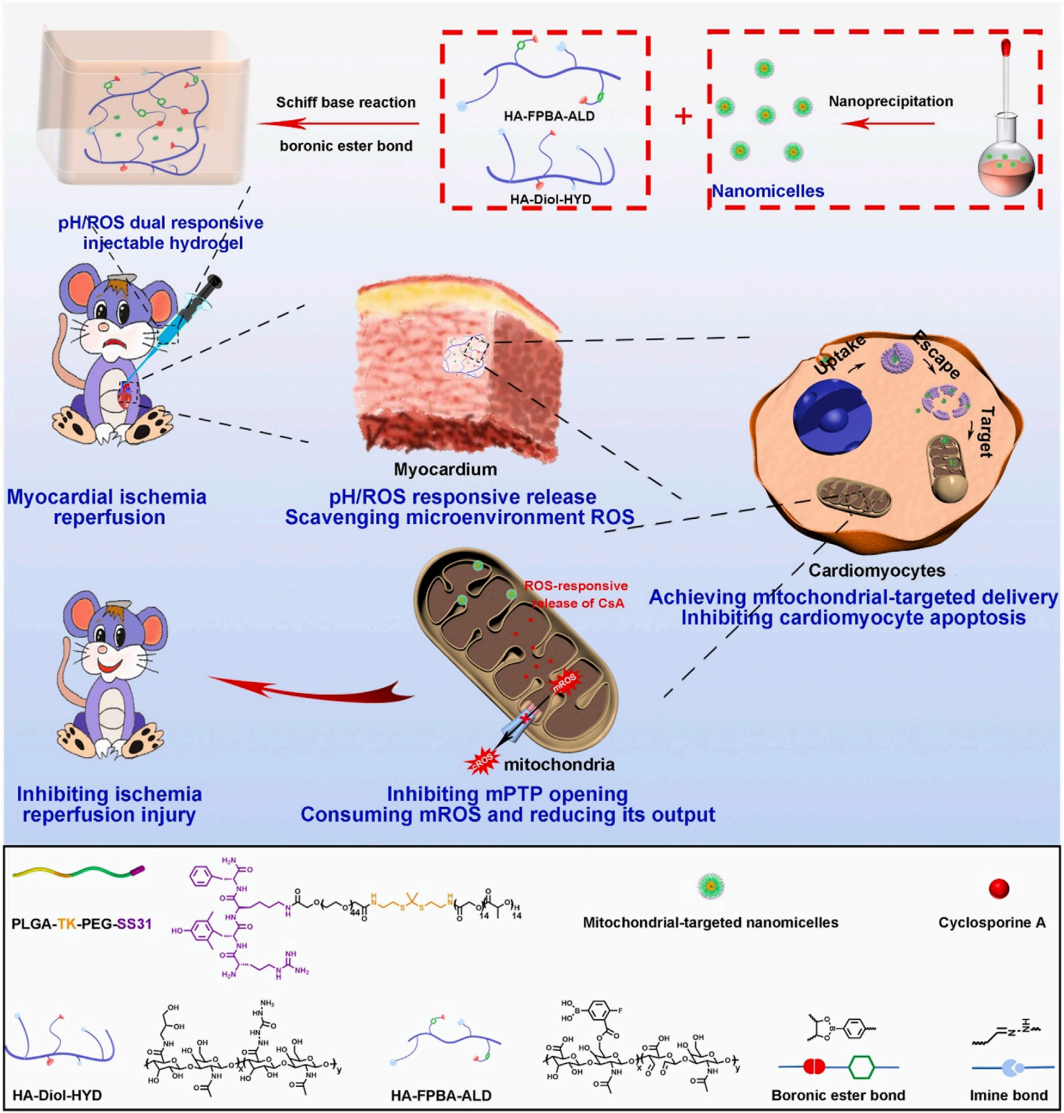
Schematic illustrating the preparation of ROS-responsive, mitochondria-targeted nanomicelles encapsulating CsA, and the construction of a pH/ROS dually responsive CsA nanomicelle-loaded injectable hydrogel using Schiff base reactions and boronic ester bonding crosslinks. In a rat model of myocardial ischemia-reperfusion injury, this hydrogel ensures precise delivery of nanodrugs to mitochondria in response to low pH and high ROS, reducing oxidative stress at microenvironmental, cellular, and subcellular levels, and restoring mitochondrial and myocardial functions. Reproduced with permission (Zhang X. et al., 2022). Copyright 2022, Elsevier.

In addition, Zhang and colleagues have devised a hierarchical targeting pH and ROS dual-responsive hydrogel system that aimed to restore mitochondrial function and alleviate oxidative stress (Zhang X. et al., 2022). Notably, this system utilized both thioketal and boronic ester linkages known for their ROS-responsive degradation properties. Initially, mitochondrial-targeting polymeric micelles (PTPSC) were constructed, which self-assemble from thioketal-crosslinked PLGA and PEG (PLGA-TK-PEG) modified with the mitochondrial-targeting Szeto-Schiller (SS31) peptide. Subsequently, these PTPSC micelles were encapsulated within a pH/ROS dual-responsive injectable hydrogel crosslinked by reversible imine and boronic ester bonds, and loaded with cyclosporine A (CsA), a well-established drug known to inhibit the opening of the mitochondrial permeability transition pore (mPTP). In response to the low pH and high ROS in cardiac tissue during reperfusion, the imine and boronate bonds in the hydrogels were broken and the CsA-loaded PTPSCs were controllably released from the hydrogel matrix into damaged cardiomyocytes (Figure 6). The elevated intracellular ROS further induced the cleavage of the thioketal linker and targeted the release of CsA into the mitochondria via SS31 peptide, thereby blocking the opening of the mPTP and inhibiting mitochondria-mediated cardiomyocyte apoptosis, while attenuating the output of mitochondrial ROS to reduce cytoplasmic ROS levels. In rat models, this novel smart hydrogel system demonstrated remarkable therapeutic efficacy by restoring mitochondrial and cardiac functions, underscoring its potent ROS scavenging capabilities and innovative contribution to cardiac repair. The integration of these state of the art biomaterials and mechanisms underscores a promising approach for targeted therapy, especially in mitigating oxidative stress-related pathologies where mitochondrial dysfunction plays a pivotal role.

### 4.3 Biomimetic biomaterials

Given the remarkable biocompatibility, low immunogenicity, and low toxicity of biomimetic nanomaterials that incorporate cells, cellular components (such as membranes, lipoproteins, etc.), and extracellular vesicles (EVs), biomimetic nanomaterials have emerged as a prominent strategy in nanomedicine development (Yaman et al., 2020). In the treatment of ischemic heart disease, this biomimetic approach enables nanomaterials to disguise themselves as endogenous substances and target damaged myocardial areas, thereby minimizing toxicity and enhancing biocompatibility (Laiva et al., 2015; Kang and Kwon, 2022). A recent systematic review comprehensively summarized the research progress on biomimetic nanomaterials based on different cell types of biomembranes and EVs for ischemic heart disease therapy (Yu et al., 2024). Notably, in recent years, research has focused on integrating various microenvironmental responsive elements into these biomimetic nanodelivery platforms to improve their drug loading targeting and controlled release capabilities (Rios et al., 2015; Wu et al., 2016; Kobayashi et al., 2018; Liu et al., 2019). In studies on alleviating MIRI, to date, only two study has used ROS-responsive polymeric materials to load anti-inflammatory and pro-suvival drugs and integrated them into platelet membrane chimeric nanodelivery system to achieve targeted drug delivery in damaged myocardium (Li et al., 2022; Weng et al., 2022).

**FIGURE 7:**
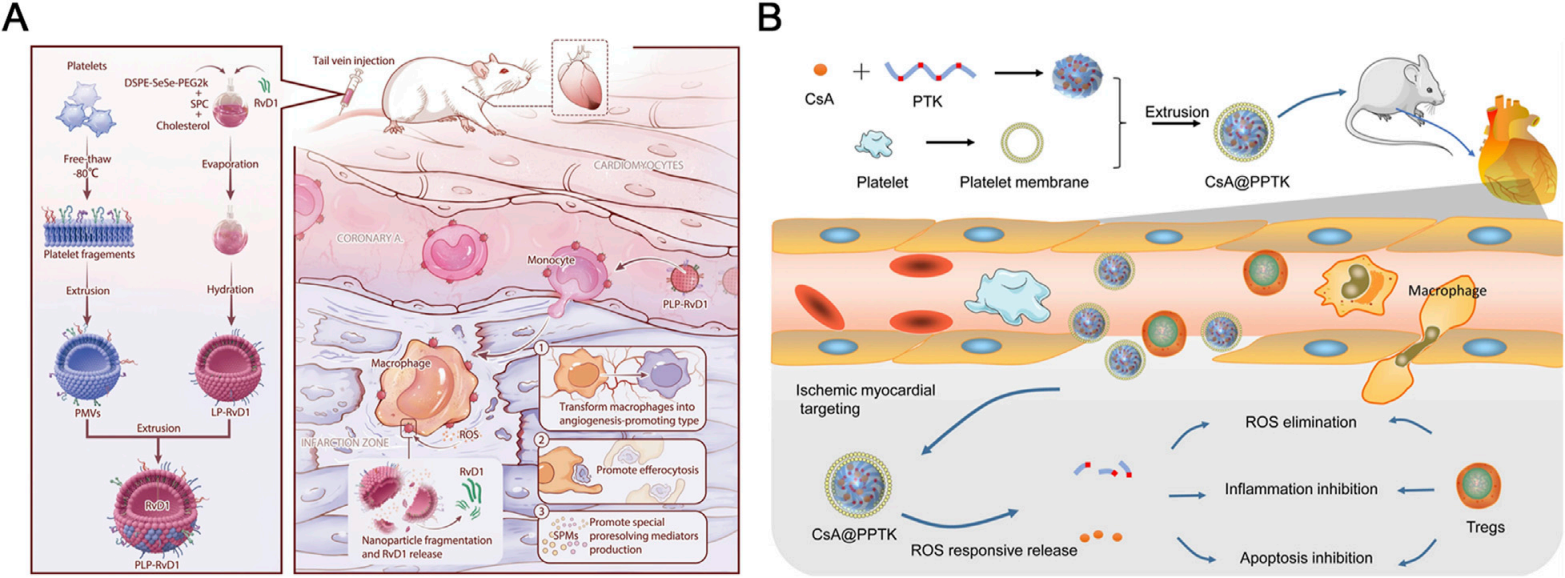
**(A)** A delivery platform called PLP-RvD1, which is responsive to ROS and mimics platelets, was designed. It is created by combining ROS-responsive liposomes loaded with RvD1 and platelet membranes. This platform retains the platelets’ ability to interact with monocytes, allowing it to reach cardiac injury sites by hitching a ride with chemotactic circulating monocytes following intravenous injection. The high levels of ROS at the injury site break down the platform, releasing RvD1 quickly. The released RvD1 then facilitates the clearance of dead cardiomyocytes, the production of SPMs, and angiogenesis, which helps improve ventricular remodeling and maintain cardiac function in mice with myocardial I/R injury. Reproduced with permission (Weng et al., 2022). Copyright 2022, BioMed Central. **(B)** CsA@PPTK effectively accumulated in the ischemic myocardium of MI/RI mice, where it significantly reduced ROS and promoted the generation of Tregs while increasing the ratio of M2 to M1 macrophages. Additionally, CsA@PPTK notably decreased cardiomyocyte apoptosis, reduced infarct size and fibrosis in the ischemic myocardium, and lowered MMP-9 protein expression while increasing CX43 protein expression in the affected tissue. These effects led to a substantial reduction in left ventricular remodeling and a marked improvement in heart function in MI/RI mice. Copyright 2022, BioMed Central.

Specifically, Weng and colleagues developed this innovative platelet-bionic ROS-responsive delivery platform, PLP-RvD1, utilizing platelet membrane chimerism modified liposomes to achieve targeted delivery to myocardial macrophages at the injury site and mediate ROS-responsive release of the anti-inflammatory drug Resolvin D1 via diselenide bonds (Figure 7A) (Weng et al., 2022). In a mouse MIRI model, the intravenous injection of PLP-RvD1 resulted in the enrichment of RvD1 in the injured myocardium, promoting macrophage efferocytosis of apoptotic cardiomyocytes, production of specialized proresolving mediators (SPMs), and angiogenesis during injury repair process, effectively improving ventricular remodeling and protecting cardiac function. Furthermore, biosafety assessment of this delivery system demonstrated that PLP-RvD1 did not induce acute inflammatory responses, exhibited no potential immune reactions, and lacked organ toxicity, suggesting its suitability for potential clinical applications.

Similarly, Li and colleagues employed a biomimetic strategy to develop a platelet membrane-cloaked nanoparticle, CsA@PPTK (Figure 7B). This nanoparticle is constructed using poly (thioketal) (PTK), a material rich in thioketal bonds that are sensitive to various types of ROS. The ROS sensitivity allows the nanoparticle to release its encapsulated anti-apoptotic drug, CsA, specifically at the site of injury where ROS levels are elevated (Li et al., 2022). Upon intravenous injection, CsA@PPTK effectively accumulated in the infarcted myocardium of MIRI mice, where it scavenged ROS and modulated the inflammatory response. This modulation was achieved by increasing the generation of regulatory T cells (Tregs) and enhancing the M2/M1 macrophage ratio. As a result, this targeted delivery and responsive release strategy significantly reduced cardiomyocyte apoptosis, decreased infarct size, and mitigated fibrosis, leading to improved cardiac function and left ventricular remodeling. Furthermore, biosafety assessments revealed that CsA@PPTK did not induce acute inflammation, immune reactions, or organ toxicity, highlighting its potential as a promising candidate for clinical applications in treating MIRI.

In addition to the above-mentioned ROS-responsive therapeutic platforms specifically designed for MIRI, recent studies have also extensively explored ROS-responsive nanomaterials with nearly identical design principles, including the same ROS-responsive elements and material types, for the treatment of myocardial infarction (MI). These nanomaterials are often loaded with drugs that possess anti-inflammatory, antioxidant, pro-angiogenic, and anti-fibrotic properties (Yao M. Y. et al., 2022; Zheng et al., 2022b; Ji et al., 2023; Sun et al., 2024; Zhang J. H. et al., 2024). Given the close similarities in the pathological processes underlying MI and MIRI, it is highly probable that the nanomedicines developed for MI could also yield significant therapeutic benefits in the treatment of MIRI. Notably, while ROS-responsive nanomaterials carrying anti-fibrotic agents have been effectively utilized in MI therapy (Ji et al., 2023; Sun et al., 2024), they have not yet been applied in the context of MIRI treatment. The future development of anti-MIRI nanomedicine may need to adopt a more holistic approach. This would involve integrating a spectrum of cardioprotective drugs with multiple therapeutic properties, aimed at providing comprehensive protection of cardiac structure and function throughout the disease progression, from acute injury to chronic remodeling.

## 5 Prospects and conclusion

ROS overproduction during I/R play a crucial role in myocardial injury, presenting both a challenge and an opportunity for innovative treatment strategies. ROS-responsive biomaterials have emerged as a promising approach to addressing I/R injury in the heart as well as other organs such as liver, kidney and brain. Despite their potential, several challenges must be overcome to translate these biomaterials from the laboratory to clinical practice. 1) One significant challenge is the integration of multifunctionality in current ROS-responsive biomaterials to address both diagnostic and therapeutic needs. The ability to combine therapy with real-time imaging could significantly improve monitoring and treatment precision for MIRI. Furthermore, integrating multiple therapeutic modalities such as antioxidant, anti-inflammatory, pro-angiogenic, and anti-fibrotic therapies may produce synergistic effects, thereby enhancing myocardial recovery and function. 2) Another critical challenge is the complexity of the myocardial I/R microenvironment, characterized by fluctuating ROS levels and diverse pathological features that vary depending on the extent of myocardial injury in each patient. This variability directly impacts the efficacy of nanomaterials. To overcome this, it is crucial to enhance our understanding of ROS biology, focusing on the spatiotemporal changes of ROS in the myocardium after I/R. Future designs should also integrate multi-stimulus-responsive capabilities that can react to additional I/R markers such as pH and inflammation. This strategy will facilitate the creation of smart-responsive systems that can dynamically adapt to these microenvironmental changes, thereby enhancing targeting precision and therapeutic efficacy. 3) With the exception of *in situ* injected biomaterials, such as hydrogels, current therapeutic polymeric or biomimetic nanoparticles injected intravenously are difficult to effectively target to the heart and are often cleared by the liver or kidneys. Therefore, the design of these materials should also incorporate strategies that enhance their affinity and retention within the damaged myocardium, possibly through the use of heart-specific peptides or antibodies. 4) Addressing the biosafety and systemic toxicity of these advanced materials is paramount. Although nanomedicine can enhance drug bioavailability and prolong circulation times, the long-term effects require thorough investigation. Understanding the pharmacokinetics, biodegradation, and metabolic profiles of these materials is essential to ensuring their safe clinical application. Preclinical studies and clinical trials should explore the efficacy of these nanomaterials in larger animal models to better evaluate their clinical potential. A focus on rigorous preclinical testing and streamlined regulatory processes will also be essential to facilitate the transition from bench to bedside. 5) Finally, the scalability of production is another hurdle that must be overcome. As these biomaterials move towards clinical application, the need for efficient manufacturing processes that maintain material integrity and functionality at scale becomes evident. This is crucial for meeting the demands of clinical practice while ensuring cost-effectiveness.

In conclusion, significant advances in ROS-responsive biomaterials, including polymeric nanoparticles, hydrogels, and biomimetic nanomaterials, offer a promising approach to treating ischemia-reperfusion injuries by enabling targeted therapeutic delivery. These biomaterials can reduce myocardial damage more effectively than current therapeutics, but challenges remain in patient variability, targeting specificity, biocompatibility, long-term safety and efficacy, and manufacturing scalability. Continued research focusing on these areas is crucial. By addressing these challenges, ROS-responsive biomaterials could revolutionize the management of ischemic conditions across multiple organ systems, significantly improving patient outcomes and paving the way for personalized medical interventions.
